# Positron Emission Tomography as a Surrogate Marker for Evaluation of Treatment Response in Patients with Desmoid Tumors under Therapy with Imatinib

**DOI:** 10.1155/2013/389672

**Published:** 2013-05-16

**Authors:** Bernd Kasper, Antonia Dimitrakopoulou-Strauss, Lothar R. Pilz, Ludwig G. Strauss, Christos Sachpekidis, Peter Hohenberger

**Affiliations:** ^1^Sarcoma Unit, ITM—Interdisciplinary Tumor Center Mannheim, Mannheim University Medical Center, University of Heidelberg, Theodor-Kutzer-Ufer 1–3, 68167 Mannheim, Germany; ^2^Clinical Cooperation Unit Nuclear Medicine, German Cancer Research Center, Im Neuenheimer Feld 280, 69120 Heidelberg, Germany; ^3^Medical Faculty Mannheim, University of Heidelberg, Theodor-Kutzer-Ufer 1–3, 68167 Mannheim, Germany

## Abstract

We used 2-deoxy-2-[^18^F] fluoro-D-glucose (FDG) positron emission tomography (PET) to evaluate patients with desmoid tumors undergoing therapy with imatinib. The study included 22 patients with progressive disease (PD) of a biopsy proven desmoid tumor treated orally with imatinib 800 mg daily. Patients were examined using PET prior to onset of therapy and during treatment. Restaging was performed in parallel using computed tomography (CT) and/or magnetic resonance imaging (MRI). Outcome of 22 evaluable patients was as follows: five patients with partial response (PR); twelve patients with stable disease (SD) accounting for 77% with non-progressive disease; five patients showed PD. A 30% decrease of the mean average standardized uptake value (SUV) of sequential PET examinations could be demonstrated; no patient demonstrated a substantial increase in SUV. Patients with PR/SD were matched to a group of nonprogressive disease and tested versus PD. The initial average SUV and SUV_max_ seem to be candidates for a response prediction with an approximate *P*-value of *0.06553* and *0.07785*, respectively. This is the first larger series of desmoid patients monitored using PET showing that early SUV changes may help to discriminate responders from nonresponders and, thus, to decide whether imatinib therapy should be continued.

## 1. Introduction

According to the World Health Organisation, desmoid tumors are defined as “clonal fibroblastic proliferations that arise in the deep soft tissues and are characterized by infiltrative growth and a tendency toward local recurrence but an inability to metastasize.” They may affect all sites including extremities, trunk, and abdomen with an incidence less than 3% of soft tissue sarcomas [1, 2]. They occur between the age of 15 and 60 years, but particularly during early adolescence and with a peak age of about 30 years. There is a special relationship between desmoids and familial adenomatous polyposis (FAP, Gardner syndrome) with an incidence from 3.5% to 32% [3, 4]. Surgical resection remains the therapeutic mainstay in first-line treatment for locally circumscribed desmoid tumors. However, R0 resection is not always possible, and adjuvant radiotherapy is, therefore, common. Due to their locally aggressive growth, desmoids have a high relapse rate after surgery and/or radiotherapy; they can often take a multiply relapsing, multifocal course and, therefore, not be amenable to curative surgical treatment. In this situation, pharmacotherapy is used to prevent disease progression comprising antihormonal therapy, nonsteroidal anti-inflammatory drugs, or chemotherapy with highly variable results [5, 6]. The primary aim is to preserve the patient's quality of life which is threatened by loss of function and pain caused by the proliferative disease. It has not yet been possible to establish an optimal therapeutic strategy or treatment algorithm for this disease.

Imatinib is a selective inhibitor of the tyrosine kinases ABL and KIT and platelet-derived growth factor receptors *α* and *β* (PDGFRA and PDGFRB) being effective in patients with Philadelphia chromosome-positive chronic myelogenous leukemia and metastatic gastrointestinal stromal tumors (GIST) [7, 8]. Initial data on the use of imatinib in desmoid tumors observed a response in two patients [9]. In desmoids, it is uncertain whether the response is due to the inhibition of known imatinib targets, and no genomic mutations have been observed showing that the response to imatinib is attributable to c-kit expression [10]. Heinrich et al. (2006) treated 19 patients with desmoid tumors with 800 mg imatinib daily; three PR and four SD were observed. Genomic analyses revealed no mutations of KIT, PDGFRA, or PDGFRB [11]. The French Sarcoma Group published a phase II study with 40 patients demonstrating one complete response and three PR at three months. The nonprogression rates at 3, 6, and 12 months were 91%, 80%, and 67%, respectively. The 2-year progression-free (PFS) and overall survival (OS) rates were 55% and 95%, respectively [25]. Chugh et al. observed similar response and nonprogression rates in 51 patients [12].

It is still questionable whether a change in tumor size is a meaningful tool for the evaluation of patients' outcome when treated with tyrosine kinase inhibitors. Standard radiographic response according to RECIST has not correlated consistently with histological response, disease-free survival, or OS. Other methods identifying patients who likely benefit from chemotherapy or other agents are needed. Therefore, ^18^F-FDG PET has found increasing use in oncology as it can visualize soft tissue tumors and detect local and distant disease recurrence in malignancies [13]. The SUV of ^18^F-FDG correlates with the metabolic rate of FDG accumulation in tumor cells [14]. Hence, the SUV could function as an easily measurable surrogate marker of tumor viability during treatment. In a group of 46 patients with localized, intermediate/high grade extremity soft tissue sarcomas, it could be demonstrated that SUV changes during neoadjuvant chemotherapy can be used to predict therapy outcome [15]. Thus, it has been suggested that ^18^F-FDG PET can act as a noninvasive method to predict patients who are less likely to benefit from doxorubicin-based chemotherapy [16].

However, no data have been published for the use of PET in desmoid tumor patients under treatment with imatinib, except of a pilot study from our group [17]. The purpose of the present study was to analyze and discuss semiquantitative ^18^F-FDG PET measurements in a collective of patients with desmoid tumors treated with imatinib.

## 2. Patients and Methods

### 2.1. Patients

The study included 22 patients with desmoid tumors with a mean age of 46.6 ± 16.4 years and a median age of 42.5 years ranging from 22 to 75 years. Patients' characteristics including gender, age, tumor site, and previous treatments are summarized in Table 1. All patients were referred to our outpatient service with the diagnosis of a desmoid tumor confirmed by histology obtained from surgical specimens. Tumor specimens were classified according to the Fédération Nationale des Centres de Lutte Contre le Cancer (FNCLCC) system [18]. The indication for patients' inclusion in the study was RECIST PD, not amenable to surgical resection with R0 intent or accompanied by an unacceptable function loss or deficit. Main exclusion criteria were prior therapy with imatinib, severe hepatic dysfunction, and prior malignancies. Patients were treated at the Mannheim University Medical Center, University of Heidelberg since May 2006. The research was carried out according to the principles set out in the Declaration of Helsinki in 1964 and all subsequent revisions.

### 2.2. Imatinib

Imatinib mesylate was supplied as 400 mg capsules that were taken orally (Novartis Pharma GmbH, Nurnberg, Germany). All patients with advanced and/or non resectable disease started imatinib therapy in a daily dose of 400 mg; treatment dose was escalated within two weeks to 800 mg daily (2 × 400 mg).

### 2.3. Imaging Studies

Patients were examined using ^18^F-FDG PET prior to onset of therapy with imatinib and during imatinib treatment. The treatment/imaging algorithm was as follows. (a) An initial PET examination was performed at baseline before start of imatinib treatment. (b) A second PET examination was done for therapy monitoring after one to three months; if SUV decreased or was stable, imatinib treatment was continued. (c) Another follow-up PET was performed in some cases for further treatment monitoring. Conventional imaging of the same target lesion using CT and/or MRI was performed in parallel to determine response according to RECIST. This data served as reference to evaluate the response determined with ^18^F-FDG PET. Dynamic PET studies were performed after intravenous injection of 300–370 MBq FDG for 60 min. A dedicated PET system (ECAT EXACT HR plus, Siemens, Erlangen, Germany) or a PET-CT system (Biograph mCT, S128) was used for patient studies as described before [19]. PET-CT studies were performed using a low-dose CT (30 mA) with current modulation without any contrast material. The CT data were used for attenuation correction and for the image fusion. The last images (55–60 minutes after injection) were used for semiquantitative analysis. PET cross-sections were reconstructed with an image matrix of 256 × 256 (for ECAT EXACT HR plus) or 400 × 400 (for Biograph mCT) using an iterative reconstruction program. Images were scatter- and attenuation-corrected. Volumes of interest (VOI) were placed over the lesion. To acquire information about the tumor viability, the hypermetabolic areas of the tumors were evaluated and hypometabolic areas that correlate to necrotic tissue were excluded. The SUV in the tumor was calculated according to the following equation: SUV = tissue concentration (MBq/g)/[injected dose (MBq)/body weight (g)]. The SUV reflected the average SUV value provided by the quantification software in VOI. This value is more robust than the maximum SUV (SUV_max_), because it is less influenced by the parameters used for the image reconstruction as well as by potential artefacts. A major limitation of the use of SUV_max_ is that it is highly dependent on the statistical quality of the images and the size of the maximal pixel and is, therefore, less robust than the use of the average SUV within VOI [20]. The analysis of the PET images was performed by two nuclear medicine physicians using a dedicated software package.

### 2.4. Statistical Analysis

Standard descriptive statistical analysis for the data was performed. PFS was defined as the time interval from the date of imatinib therapy induction until tumor progression, end of therapy, or data acquisition. Parameters for PFS and SUV were given as mean and median with range. Skewness for SUV1 and SUV1_max_ was higher than for the other variables. For calculated ratios and differences of the variables, the facts are so far different, since the differences show a greater skewness and the ratios are near normal distributed meaning that the Wilcoxon rank sum test will be suitable for the differences but not the *t*-test. For descriptive statistical analysis, StatXact-9 of Cytel Studio, Version 9.0.0, Cytel Inc., Cambridge, MA, USA, and for the tests the SAS software 9.2 (TS2M3) by the SAS Institute Inc., Cary, NC, USA, were used.

## 3. Results

### 3.1. Clinical Response Based on RECIST Criteria

Imatinib was taken orally in a dose of 800 mg daily. The therapy interval with imatinib was in the mean 19.7 months with a median of 12.5 months (range: 1–74) until time of data acquisition. In spite of CTCAE grade I/II fatigue and edema, no major (grade III/IV) toxicities occurred. First CT and/or MRI scan was performed in all patients prior to onset of therapy with imatinib. Restaging was performed using CT and/or MRI every three months after start of imatinib treatment. The remission status was evaluated according to RECIST based on the tumor shrinkage in the CT and/or MRI scan. Clinical outcome according to RECIST was as follows: five patients with PR (23%), 12 patients with SD (55%), and five patients with PD (23%). The mean PFS from the date of therapy induction until end of therapy or data collection for all patients was 20.6 months with a median of 14 months ranging from 1 to 74 months. The six-month PFS rate was 68%, and all patients are alive at the time of data acquisition.

### 3.2. Clinical Response Based on PET Imaging

**Figure 1:**
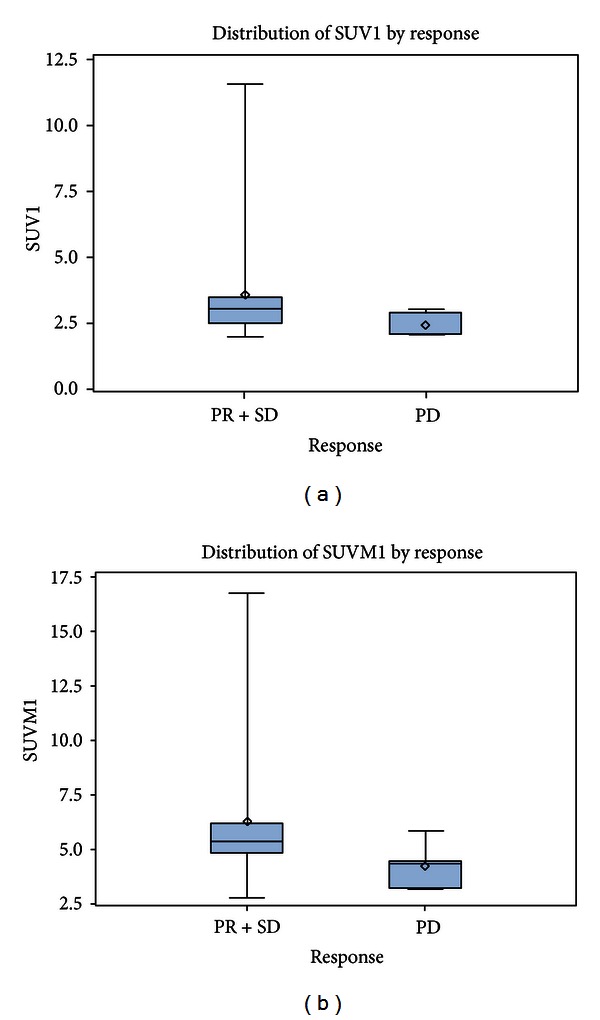
The box plots show the distribution of the average SUV1 and SUV1_max_ values by conventional response evaluation according to RECIST criteria for the group of nonprogressive patients (PR + SD) versus patients with PD with an approximate *P* value of *0.06553* and *0.07785*, respectively.

In all patients, two sequential PET examinations have been performed within a median time interval of 53.5 days; in 13 patients, more than two PET examinations were done during imatinib treatment within a median time interval of 199.5 days from the baseline PET. The median average SUV prior to onset of targeted therapy with imatinib was 2.9 (range: 2.0–11.6) in comparison to 2.1 (range: 1.5–3.4) during treatment. The median SUV_max_ was 5.1 (range: 2.8–16.8) prior to therapy with imatinib in comparison to 4.1 (range: 2.3–6.1) following treatment. Hence, a decrease of 28% of the median average SUV and a decrease of 20% of the median SUV_max_ for sequential PET examinations could be demonstrated for the evaluated patients; no patient demonstrated a substantial increase in SUV. However, the main question was whether the PET results can predict response evaluation by conventional RECIST criteria and, thus, act as a surrogate marker. Therefore, the initial SUV and SUV_max_ (SUV1 and SUV1_max_) were used as a basis for multiple testing in the three categories PR, SD, and PD in comparing these with the data of the second or third PET examination if available. Multiple testing was performed with the multiple Wilcoxon rank sum test and the multiple *t*-test; however, none of the tests were significant. In a second approach, patients with PR and SD were matched to a group of nonprogressive disease and tested versus patients showing PD. Using the Wilcoxon rank sum test, SUV1 and SUV1_max_ seem to be candidates for a response prediction with an approximate *P* value of *0.06553* and *0.07785*, respectively, (Figure 1). In the literature, for soft tissue sarcomas in general, a cut-off value of 40% SUV reduction from baseline has been chosen to differentiate responders [21]. In our collective, four patients demonstrated an at least 40% SUV decrease, three of them showing SD and one PR (*compare* Table 2), whereas the other patients showed stabilization or an SUV decrease of less than 40%.

### 3.3. Patient Example

**Figure 2:**
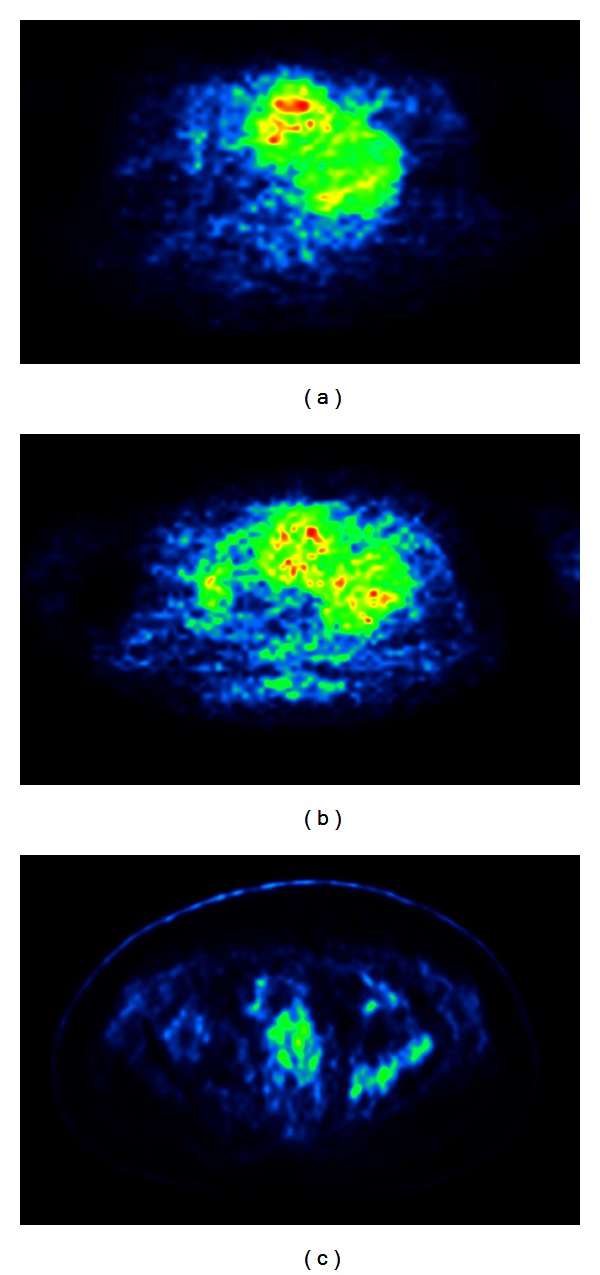
A 31-year-old female with a retroperitoneal desmoid tumor (case 3, Table 2; Figure 2) diagnosed in 2006 was treated with imatinib 800 mg daily. The FDG PET prior therapy with imatinib showed an average SUV of 4.2 and an SUV_max_ of 8.1 (a). After one month of imatinib treatment, the FDG PET demonstrated a decrease of the average SUV to 3.3 (−22%) and of the SUV_max_ to 6.1 (−25%) (b). Follow-up PET examinations in 2011 and 2012 (c) did not show any pathological FDG uptake. The corresponding conventional MRI documented PR according to RECIST.

A 31-year-old female with a retroperitoneal desmoid tumor (case 3, Table 2; Figure 2) diagnosed in 2006 was treated with imatinib 800 mg daily. The FDG PET prior therapy with imatinib showed an average SUV of 4.2 and an SUV_max_ of 8.1. After one month of imatinib treatment, the FDG PET demonstrated a decrease of the average SUV to 3.3 (−22%) and of the SUV_max_ to 6.1 (−25%). Follow-up PET examinations in 2011 and 2012 did not show any pathological FDG uptake.The corresponding conventional MRI documented PR according to RECIST.

## 4. Discussion

There are different implications for the use of PET in soft tissue tumors. It has been studied to predict the malignant potential and grading, to stage the malignant disease, to monitor tumor response and predict clinical benefit from chemotherapy [22]. However, most of the studies comprised only small numbers of patients using different imaging protocols and evaluation procedures making comparison extremely difficult. Changes in tumor size to chemotherapeutic treatment have been the parameter to predict the therapeutic benefit for the patients. However, changes in tumor size measured with CT and/or MRI did not correlate consistently with sarcoma patients' outcomes. For GIST, this finding has already been well documented: a study of ^18^F-FDG PET in imatinib treated GIST showed that patients with normalization of the SUV within the first month of treatment have a significantly longer time to disease progression and better OS than those patients with increased ^18^F-FDG accumulation [23]. ^18^F-FDG PET appears to be more useful than CT/MRI imaging in GIST to predict therapy response. Moreover, there is even doubt if RECIST criteria adequately describe the remission status to chemotherapy or other targeted agents. Therefore, a new classification of response criteria, “(PERCIST) Positron Emission tomography Response Criteria In Solid Tumors,” has been introduced taking into consideration both changes in tumor volume as well as changes in metabolism [24].

To our knowledge, the present paper describes the first larger series of desmoid tumor patients under therapy with imatinib monitored with sequential PET imaging despite a pilot study presented from our group [17]. In our patient population, a significant SUV decrease (≥ 40%) of sequential PET examinations could be demonstrated in four patients (18%), whereas the other patients showed stabilization or an SUV decrease of less than 40%. There was no patient in this series demonstrating a substantial SUV increase. Considering the fact that patients had to demonstrate RECIST PD to enter the study, the high proportion of 77% of patients with nonprogressive disease means a significant benefit. RECIST criteria seem inadequate to describe responses seen in patients with desmoid tumors. Complete or even PR are documented in the literature in around 10% of patients treated with imatinib [11, 12, 25]; in our series with 23% the response rate was relatively high. Most of the patients show disease stabilization or even shrinkage of the tumor. However, considering the fact that patients were inoperable or demonstrated PD at the time entering the study, control of symptoms and disease stabilization mean a substantial clinical benefit for most of the patients initially suffering from pain or functional loss. Therefore, benefit can be defined for most of the patients as a progression arrest.

The characteristics of imatinib treatment in desmoid tumor patients seem to be confirmed by PET: imatinib has a remarkable ability to slow the growth and stabilize the tumor. Of course, compared to high-grade soft tissue sarcomas, baseline SUV values are relatively low in desmoid tumors (initial median average SUV of 2.9). Therefore, documented SUV changes under treatment with imatinib were relatively small. We could show that PET monitoring of desmoid patients under treatment with imatinib may be used to determine whether patients benefit from imatinib therapy or not in the lack of an adequate CT and/or MRI imaging [26]. In particular the initial average SUV1 and SUV1_max_ data seem to be candidates for a response prediction and may act as surrogate markers. Figure 1 shows that the higher initial average SUV1 and SUV1_max_ data are obviously associated with a higher probability of treatment response in the PR/SD versus PD proportion of patients. Therefore, we have shown that early SUV changes may be detected helping to discriminate responders from nonresponders and, thus, to decide whether imatinib therapy should be continued or not. For example, therapy with imatinib would be continued if an SUV decrease or stabilization is documented. However, if there is a substantial SUV increase, continuation of imatinib treatment is questionable having also an impact on treatment costs.

In summary, PET will certainly play an increasingly important prognostic and predictive role in the management of “semimalignant” and malignant soft tissue tumors [27–29]. It could be used to characterize the aggressiveness of the tumor in order to make clinical decisions whether treatment is useful for the patients or not. Our present data suggest that the ability of imatinib treatment to slow down the growth of desmoid tumors—resulting in a 77% progression arrest rate—is reflected by SUV stabilization or a SUV decrease of up to 83%. Furthermore, PET imaging may be used as a surrogate marker in order to predict response to therapy early in the course of treatment for cytotoxic chemotherapy and other targeted agents like sorafenib [30]. However, more data have to be evaluated to demonstrate statistically significant results.

## Figures and Tables

**Table 1:** Patients' characteristics (*n* = 22).

Gender	
Female	16
Male	6
Age	
Median (years)	42.5 (range: 22–75)
Histology	
Desmoid tumor	22
Tumor site at initial diagnosis	
Abdomen/trunk	17
Extremities	5
Previous treatment	
None	8
Surgery alone	7
Surgery plus radiotherapy	7
Systemic treatment	2

**Table 2:** PET results for desmoid patients (*n* = 22) treated with imatinib.

Patient no.	Age (years)	Tumor localization	Imatinib treatment duration (months)	Average SUV (initial)	Average SUV (follow-up)	SUV change (%)	Response according to RECIST	PFS (months)
1	64	Chest	6	2.902	2.538	−13	PD	6
2	70	Pelvis	5	3.171	3.364	6	SD	5
3	31	Retroperitoneal	74	4.233	3.294	−22	PR	74+
4	42	Mesenterium	4	3.023	2.793	−8	PD	4
**5**	**22**	**Chest**	**15**	**3.320**	**1.711**	−48	**SD**	**15**
6	35	Supraclavicular	6	2.115	1.851	−12	SD	6
7	27	Upper limb	58	3.112	2.428	−22	SD	58+
8	70	Buttock	60	2.785	2.632	−6	SD	60+
9	38	Pelvis	49	2.376	1.735	−27	SD	49+
10	68	Shoulder	12	2.098	1.458	−31	PD	12
11	43	Upper limb	8	2.100	1.600	−24	PD	8
12	48	Pelvis	9	2.900	2.800	−3	SD	9+
**13**	**40**	**Pelvis**	**26**	**3.500**	**2.100**	−40	**PR**	**26+**
14	47	Upper limb	28	2.300	1.800	−22	PR	28+
**15**	**54**	**Chest**	**1**	**5.229**	**3.100**	−41	**SD**	**22**
16	30	Pelvis	18	3.074	2.400	−22	SD	18+
**17**	**24**	**Mesenterium**	**17**	**11.573**	**1.981**	−83	**SD**	**16+**
18	48	Chest	3	2.059	1.975	−4	PD	3
19	41	Buttock	15	2.000	2.100	5	PR	15+
20	70	Parascapular	13	2.900	2.114	−27	PR	13+
21	39	Fossa ischiorectalis	5	2.492	2.100	−16	SD	5+
22	75	Mesenterium	1	4.037	n.e.	n.e.	SD	1+

SUV: standardized uptake value; RECIST: Response Evaluation Criteria in Solid Tumors; PFS: progression-free survival; PR: partial response; PD: progressive disease; SD: stable disease; n.e.: not evaluable; +: patient is still progression-free at the time of data collection and continues treatment with imatinib.
